# Fragment-Based Local Coupled Cluster Embedding Approach for the Quantification and Analysis of Noncovalent Interactions: Exploring the Many-Body Expansion of the Local Coupled Cluster Energy

**DOI:** 10.1021/acs.jctc.1c00005

**Published:** 2021-05-26

**Authors:** Soumen Ghosh, Frank Neese, Róbert Izsák, Giovanni Bistoni

**Affiliations:** Max-Planck-Institut für Kohlenforschung, Kaiser-Wilhelm-Platz 1, D-45470 Mülheim an der Ruhr, Germany

## Abstract

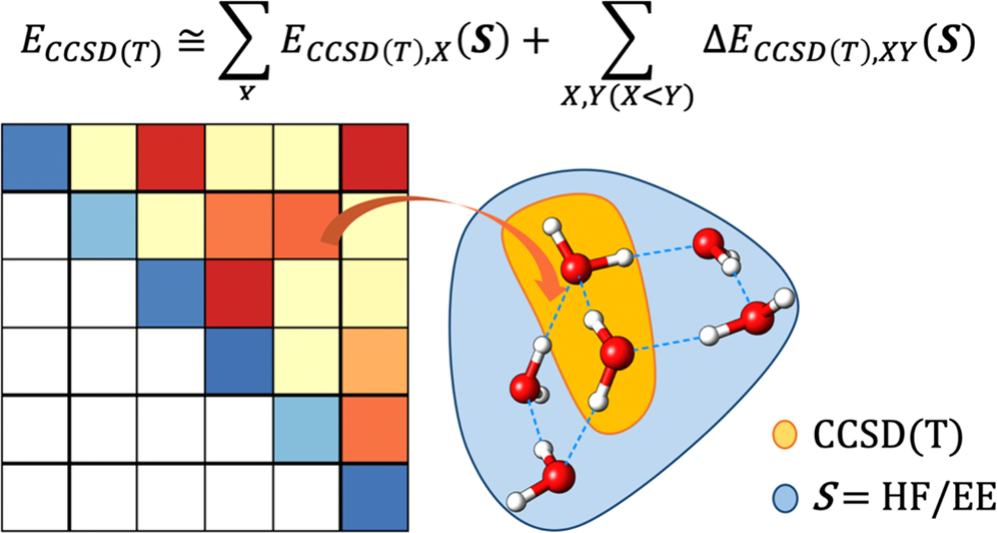

Herein, we introduce a fragment-based local coupled cluster embedding approach for the accurate quantification and analysis of noncovalent interactions in molecular aggregates. Our scheme combines two different expansions of the domain-based local pair natural orbital coupled cluster (DLPNO-CCSD(T)) energy: the many-body expansion (MBE) and the local energy decomposition (LED). The low-order terms in the MBE are initially computed in the presence of an environment that is treated at a low level of theory. Then, LED is used to decompose the energy of each term in the embedded MBE into additive fragment and fragment-pairwise contributions. This information is used to quantify the total energy of the system while providing at the same time in-depth insights into the nature and cooperativity of noncovalent interactions. Two different approaches are introduced and tested, in which the environment is treated at different levels of theory: the local coupled cluster in the Hartree–Fock (LCC-in-HF) method, in which the environment is treated at the HF level; and the electrostatically embedded local coupled cluster method (LCC-in-EE), in which the environment is replaced by point charges. Both schemes are designed to preserve as much as possible the accuracy of the parent local coupled cluster method for total energies, while being embarrassingly parallel and less memory intensive. These schemes appear to be particularly promising for the study of large and complex molecular aggregates at the coupled cluster level, such as condensed phase systems and protein–ligand interactions.

## 1 Introduction

Noncovalent interactions (NCIs) play an important role in all fields of chemical research, determining the formation of intermolecular complexes, the structure and properties of large biomolecules, and the properties of condensed phase systems. Two types of computational approaches—perturbative and supermolecular—can be used to study NCIs in large and complex systems of many interacting molecules.

Among the perturbative approaches, symmetry-adapted perturbation theory (SAPT)^1−5^ has found many applications in the study of weak NCIs due to its great accuracy and the fact that the various perturbation terms can be easily interpreted chemically.^6,7^ Unfortunately, in most implementations, SAPT is only applicable to intermolecular interactions between a pair of monomers in a dimer. Going beyond dimers within the SAPT framework, although possible theoretically,^8−10^ would necessarily increase the computational cost of the method, limiting its applicability to relatively small systems.^11,12^ Hence, to tackle larger systems while remaining within the SAPT framework, one needs to devise practical approximations to the induction and dispersion terms, as it is done in the recently developed XSAPT approach.^13−15^ In the most recent implementations of this method, the zeroth-order wave function is defined as the product of the monomer wavefunctions at the semiempirical XPol level,^16^ while the dispersion energy is replaced with an atom-pairwise empirical dispersion correction.

Within a supermolecular approach, NCIs can be computed as the energy difference between a molecular aggregate (also called “supersystem”) and its constituting monomers. Electronic structure methods of different accuracy and computational cost can be used to compute the individual energies. For example, the “gold standard” coupled cluster method with singles, doubles, and perturbatively included triple excitations, CCSD(T),^17^ is considered to be the most accurate computational method for studying NCIs among those applicable to systems of medium size. Unfortunately, CCSD(T) in its canonical implementation scales as *O*(N^7^) with the system size, which significantly limits its applicability to relatively small systems with a few hundred basis functions.

A possible strategy to avoid the inherent steep scaling of accurate electronic structure calculations relies on the many-body expansion (MBE) of the total energy of the supersystem.^18−22^ Within the MBE, the energy of the supersystem is expressed exactly as a sum of contributions from all possible monomers (one-body), dimers (two-body), trimers (three-body), etc. Hence, the calculation on the supersystem is reduced to a series of smaller, independent calculations on the subsystems, making the method embarrassingly parallel. Of course, the MBE needs to be truncated in practical applications, and the accuracy of truncated MBEs depends on the nature of the NCIs in the system.^23,24^

In particular, a defining feature of NCIs is that their strength and nature may change drastically in the presence of other NCIs.^25,26^ This property is sometimes termed “cooperativity” and plays an important role in a large variety of systems, including biomolecules,^27−29^ van der Waals complexes of π-systems,^30^ and hydrogen^31^ and halogen-bonded^32^ adducts. In the MBE framework, a “strong” cooperativity manifests itself in large contributions to the energy from the terms of order higher than 2.^22^ Clearly, truncated MBEs are not accurate for systems where such “many-body” effects play an important role.

To overcome this limitation, embedding can be used to accelerate the convergence of the MBE by effectively incorporating many-body effects into the one- and two-body terms.^18,19,33−48^ Hence, the low-order terms are computed in the presence of the remaining molecules (denoted hereafter as the “environment”), which are treated at a lower level of theory. Kitaura and co-workers first proposed the fragment molecular orbital (FMO) method, in which terms are added to the Hamiltonians of the fragments and dimers to account for the electrostatic potential of the environment. Then, the densities of the fragments and dimers are converged self-consistently.^33,35^ Later, the effective fragment molecular orbital (EFMO) approach was introduced.^49^ This method combines the FMO method with an effective fragment potential (EFP) force field. In addition, a series of electrostatically embedded MBE (EE-MBE) approaches have been proposed by Truhlar and co-workers, in which the environment is represented by point charges computed at a low level of theory.^36,41^ A more sophisticated approach was introduced by Manby and co-workers, who combined the use of atom-centered spherical gaussian charges to represent the electrostatic potential of the environment with an empirical “exchange-repulsion” potential to account for the repulsive interactions between the environment and the subsystems.^37^ Quantum embedding approaches have also been proposed, in which different levels of electronic structure theory are used to model the environment,^19,40,45−48,50^ such as the popular wavefunction-in-DFT embedding method developed by Manby, Miller, and co-workers.^48,50^ Moreover, the recently proposed embedded mean-field theory^51^ (EMFT) of Manby, Miller, and co-workers, which permits the use of different mean-field approaches for the fragments and the environment, was recently used by Head Gordon and co-workers as a basis for the development of a “polarized MBE” quantum embedding scheme, which was applied to the calculation of binding energies in small molecular clusters.^19^ Finally, the cluster-in-molecule (CIM) method is a quantum embedding scheme that is based on localized molecular orbitals, which can be used to accelerate the calculation of correlation energies obtained with post Hartree–Fock (HF) methods.^52,53^ To reduce the cost of the underlying HF calculations, the FMO approach has also been combined with CIM.^54^

In this work, we present an approach to quantify and analyze NCIs accurately and in great detail, which is based on an embedded MBE of the CCSD(T) energy of the supersystem. In particular, our approach relies on the domain-based local pair natural orbital variant of the CCSD(T) method, that is, DLPNO-CCSD(T).^55−61^ A large number of benchmark studies demonstrated that the DLPNO-CCSD(T) method can be used to compute relative energies for systems with hundreds of atoms and thousands of basis functions with great accuracy.^60,62,63^

In our method, the well-established local energy decomposition (LED) analysis^63−66^ is used to decompose exactly the low-order terms in the embedded MBE of the DLPNO-CCSD(T) energy of the supersystem into a series of additive contributions corresponding to: (i) the energy of the embedded monomers; (ii) the interaction energy between pairs of embedded monomers (for the terms of order higher than 1); (iii) the energy of the environment; and (iv) the interaction energy between the environment and each of the monomers. This information is used to quantify the total energy of the supersystem, leading to an embarrassingly parallel method. Moreover, an in-depth, term-by-term comparison between the decomposed contributions of different orders provides unique insights into the nature of cooperativity in noncovalent interactions.

Two different approaches are tested: the local coupled cluster in the Hartree–Fock (LCC-in-HF) embedding scheme, in which the one- and two-body terms in the MBE are treated at the DLPNO-CCSD(T) level in the presence of an Hartree–Fock (HF) environment; and the local coupled cluster in an electrostatic embedding (LCC-in-EE) method, in which the environment is replaced by a series of point charges.

The article is organized as follows. In Sections 2.1 and 2.2, we recall the basic principles of the MBE and of the LED approaches, respectively. In Section 2.3, it is shown how the LED and MBE schemes can be combined to study cooperativity of NCIs. Besides being a valuable interpretation tool for the analysis of NCIs in chemical applications, the combination of MBE and LED allows us to thoroughly test the accuracy of the embedded MBE approaches proposed here. In Sections 2.4 and 2.5, the LCC-in-HF and the LCC-in-EE methods are introduced, respectively, while computational details are given in Section 2.6. In Section 3.1, the LCC-in-HF and the LCC-in-EE methods are tested on a series of water clusters, which are known to be challenging for local coupled clusters techiques^60,62,63^ and to feature large many-body effects.^41,67,68^ Initially, the optimal DLPNO-CCSD(T) settings for H-bonded systems are determined (Section 3.1.1) and used to obtain accurate reference data. These results are then used to discuss the accuracy of the LCC-in-HF and the LCC-in-EE approaches (Section 3.1.2). In Section 3.2, the accuracy and efficiency of these methods are further discussed on a larger example, namely, the interaction of the imidacloprid ligand with a nicotinic acetylcholine receptor (nAChR) model.^69^

## 2 Computational Details and Theoretical Aspects

### 2.1 Many-Body Expansion (MBE)

For a system of *N* interacting fragments, the total energy can be exactly expressed using the MBE as a sum of one-body (*E*^1^), two-body (Δ*E*^2^), three-body (Δ*E*^2^), ..., N-body terms (Δ*E*^*N*^)
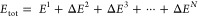
1where
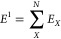
2
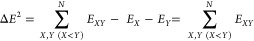
3and so on, where *E*_*X*_ is the energy of the *X*th monomer and *E*_*XY*_ is the energy of the *XY* dimer. Hence, Δ*E*_*XY*_ represents the interaction energy of the *XY* dimer.

For example, the total binding energy (also called interaction energy or association energy) of the system, Δ*E*
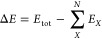
4can be written using the MBE as
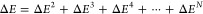
5As it was previously suggested in ref^22^, it is useful to define the overall “cooperativity effect” Δ*E*_coop._ as
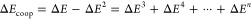
6Hence, Δ*E*_coop_ denotes the contribution to the total binding energy Δ*E* originating from the many-body terms.

### 2.2 Local Energy Decomposition of the DLPNO-CCSD(T) Energy

An alternative decomposition of the total energy is provided by the LED scheme in the DLPNO-CCSD(T) framework.^63−66^ By assigning the localized occupied orbitals onto the fragments in which they are dominantly localized, *E*_tot_ in eq 1 can be decomposed exactly into fragment-pairwise contributions
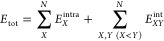
7where *E*_*X*_^intra^ represents the energy of monomer *X* when its electronic structure is perturbed by the presence of all other monomers. It is obtained by summing the contributions to the DLPNO-CCSD(T) energy associated with particles (nuclei and electrons) assigned to fragment *X*.^63−66^*E*_*XY*_^int^ represents the interaction between monomers *X* and *Y* in the presence of all other monomers. Notably, using LED, the electrostatics, exchange, and dispersion contributions to *E*_*XY*_^int^ can also be quantified.^63−66^

Using eq 4, we can now rewrite Δ*E* as
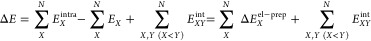
8where Δ*E*_*X*_^el-prep^ is called “electronic preparation energy” and determines the energy required to change the electronic structure of monomer *X* from its ground state to the one that is optimal for the interaction with all of the other monomers. This is by definition a positive (repulsive) contribution to the binding energy.^63−66^

### 2.3 Local Energy Decomposition of the MBE of the DLPNO-CCSD(T) Energy

In this section, a theoretical framework is presented for the accurate quantification and analysis of cooperativity effects, which combines the MBE with the LED approach. The LED scheme can be used to decompose all of the terms of the MBE of eq 1. In particular, the two-body term Δ*E*^2^ can be decomposed using eq 8 as
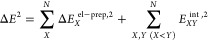
9where Δ*E*_*X*_^el-prep,2^ is the total two-body electronic preparation of monomer *X*, defined as
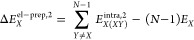
10in which *E*_*X*(*XY*)_^intra,2^ is the energy of monomer *X* when its electronic structure is perturbed by the presence of monomer *Y*, while *E*_*XY*_^int,2^ is the interaction between *X* and *Y*. By subtracting eqs 8 and 9, it is possible to write
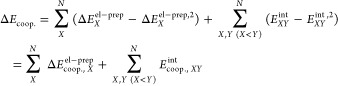
11where Δ*E*_coop., *X*_^el-prep^ represents the change in the energy of monomer *X* due to cooperativity effects. Similarly, *E*_coop., *XY*_^int^ represents the contribution of cooperativity to the interaction between monomers *X* and *Y*. To summarize, by combining the LED and the MBE schemes, one can decompose the total binding energy Δ*E*, its two-body contribution Δ*E*^2^, and the total cooperativity effect Δ*E*_coop._ into additive fragment and fragment-pairwise contributions, providing unique insights into the interplay of various noncovalent interactions.

In the following, all of these components are presented as heat plot matrices where the diagonal elements represent electronic preparation energies while off-diagonal elements represent interactions between pairs of fragments. These plots are denoted as “LED interaction maps”.^69^ As it will be demonstrated below, this type of analysis appears to be particularly useful for testing the performance of novel embedding schemes aimed at recovering many-body effects accurately in truncated MBEs.

### 2.4 Local Coupled Cluster-in-HF Approach

The theory behind the DLPNO-CCSD(T) methodology has already been discussed in detail in a series of publications.^55−61^ An important feature of this method is the so-called “multilevel” approach, which can be used to treat different parts of the system at different levels of theory.^70^

Herein, the multilevel implementation of the DLPNO-CCSD(T) method is used to define a polarized MBE quantum embedding approach, in which the one-body and the two-body components of the energy (eq 1) are computed at the DLPNO-CCSD(T) level of theory in the presence of an HF environment (which is treated as an additional fragment). The interaction between the environment and the dimer is also treated at the HF level. A smaller basis set can be used for the environment to increase the efficiency of the approach. Thus, for each pair of fragments, the coupled cluster equations are initially solved for the embedded system. Then, the LED scheme is used to extract the DLPNO-CCSD(T) components of the energy, while the energy of the environment and its interaction with the DLPNO-CCSD(T) subsystem is neglected. From here onwards, this approach is denoted as the local coupled cluster-in-HF (LCC-in-HF) method. If different basis sets, *e.g*., basis1 and basis2, are used for the subsystem and the environment, respectively, the method is denoted as LCC(basis1)-in-HF(basis2).

For example, LCC-in-HF can be used to compute approximate binding energies (eq 4) using the following steps:

- **Step 1**. Compute the energy of all of the isolated monomers *E*_*X*_ at the DLPNO-CCSD(T) level;
- **Step 2**. Compute the energy of the monomers in the presence of the HF embedding *E*_*X*_(***S***) using the multilevel implementation, where ***S*** denotes the environment. Using LED, we obtain approximate electronic preparation energies
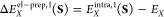
12where the superscript “1” is used to indicate quantities obtained using the embedded one-body approximation.
- **Step 3**. Compute the energy of the dimers in the HF embedding using the multilevel implementation: *E*_*XY*_(***S***). Using LED, we obtain a series of embedded interfragment contributions *E*_*XY*_^int,2^ (***S*****)**, where the superscript “2” indicates quantities computed within the embedded two-body approximation. Moreover, we also obtain a series of intrafragment energies *E*_*X*(*XY*)_^intra,2^ (***S***), which correspond to the DLPNO-CCSD(T) energies of fragment *X* when the dimer *XY* is treated at the DLPNO-CCSD(T) level and all other fragments are described by the HF environment. This can be used to compute the final electronic preparation energies Δ*E*_*X*_^el-prep, 2^ (**S**)
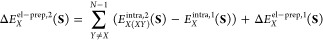
13
- **Step 4**. The total binding energy is then obtained as
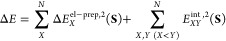
14
  More in general, the expression for the total LCC-in-HF energy of a system of many interacting molecules reads
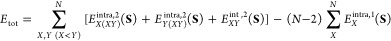
15

There are some important points that deserve to be discussed in more detail:

(i) LCC-in-HF avoids the computationally demanding (in terms of memory and computational time) DLPNO-CCSD(T) calculation on the full system by performing a series of less-expensive DLPNO-CCSD(T) calculations on the embedded monomers and dimers, which can be run in parallel.
(ii) As it will be demonstrated numerically in Sections 3.1 and 3.2, the LCC-in-HF method retains to a large extent the accuracy of the parent DLPNO-CCSD(T) method.
(iii) The LCC-in-HF method can be used to compute the interaction between specific pairs of fragments in an environment accurately, *e.g*., the interaction between a ligand and a residue in the active site of a protein or that between two species in a prereactive complex in solution. An example of such applications is given in Section 3.2.
(iv) If the same basis set is used for the subsystem and for the HF environment in LCC-in-HF, the same reference orbitals can be used for computing all of the one-body and two-body contributions, *i.e*., the HF calculation on the whole system needs to be carried out only once. In this case, eq 15 can be rewritten as
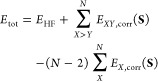
16
  where *E*_HF_ is the HF energy of the system and *E*_*XY*,corr_(**S**) and *E*_*X*,corr_(**S**) are the correlation components of *E*_*XY*_(**S**) and *E*_*X*_(**S**), respectively. This allows us to skip the LED part of the calculation, thus providing a small but often noticeable saving of computational time (∼10%). The downside is that by switching off LED, we lose the analysis of the NCIs in the system. It is also worth mentioning that the approach exemplified by eq 16 conceptually resembles the cluster-in-molecules DLPNO-CCSD(T) method (CIM-DLPNO-CCSD(T)^71^) but with some important differences. In CIM-DLPNO-CCSD(T), the total correlation energy *E*_cor_ is expressed as a sum of contributions from the localized MOs *E*_*i*,cor_
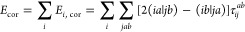
17
  where indexes *i* and *j* denote occupied localized MOs; *a*, *b* virtual orbitals; and τ_*ij*_^*ab*^ cluster amplitudes. The summation over *j* is truncated in such a way so as to include only localized MOs that are spatially close to *i*, and the amplitudes τ_*ij*_^*ab*^ associated with different *E*_*i*, cor_ contributions are not coupled.

### 2.5 Local Coupled Cluster-in-EE Method

The LCC-in-HF results will be compared with those obtained using electrostatically embedded approaches, in which the HF environment is replaced by point charges computed at the HF level of theory. Hereafter, these methods are denoted as LCC-in-EEX(PC_type*α), where “PC_type” identifies the type of point charges used, α is a scaling factor, *X* = 1 indicates that point charges are obtained from single point calculations on the whole system, and *X* = 2 indicates that point charges are obtained from separate calculations on the individual fragments. The approach with *X* = 2 has the advantage that allows us to skip the HF/aDZ calculation on the whole system. As an example, LCC-in-EE2(NPA/aDZ*1.2) denotes the use of natural population analysis (NPA) charges^72^ computed for the individual fragments at the HF/aDZ level and scaled by a factor of 1.2. The optimal PC_type/α combination must be determined for each specific application, *e.g*., by means of benchmark studies on small model systems.

In the LCC-in-EEX approaches defined here, the total energy of the system is computed using eq 15. Hence, these approaches are not equivalent to the previously published EE-MBE schemes.^68^ One important difference is that, by construction, the *E*_*X*_^intra,1^(**S**) and *E*_*X*(*XY*)_^intra,2^(S) terms do not incorporate the interaction between the electrons and the environment. In fact, they represent the energy of the isolated monomers when their electronic structure is distorted in the presence of the embedding. The second important difference is that the total energy in this approach can be decomposed without any additional computational cost into a series of additive contributions (*e.g*., dispersion and electrostatics^63−66^), thus providing new information into the nature of noncovalent interactions in the system. For the sake of simplicity, this further decomposition is not discussed in the article but illustrative examples are reported in the Supporting Information (SI) (Figure S4).

Consistent with other electrostatically embedded approaches, the accuracy of LCC-in-EEX schemes depends on the actual point charges used for the embedding. The optimal choice might change depending on the nature of the system considered, and defining a universal set of point charges for general applications goes beyond the scope of the present work. This aspect is currently under investigation in our group. However, the results reported below on water clusters and protein–ligand interactions show that LCC-in-EE1(NPA/aDZ*1.16) and LCC-in-EE2(NPA/aDZ*1.25) provide errors in NCI energies that are typically below 1 kcal/mol (see Section 3.1.2). Importantly, the LED interaction maps discussed in Section 2.3 provide in-depth information that can be used for testing the accuracy of the various embedding approaches, thus aiding to the development of accurate LCC-in-EEX schemes specifically tailored for any given application.

### 2.6 Computational Details

All calculations were performed using a development version of the ORCA suits of programs based on version 4.2.^73,74^ For LCC-in-HF and LCC-in-EE calculations, a Python program interfaced to ORCA was used to generate the many input files needed for the embedded MBE calculations as well as for data analysis.

For water clusters, all single point DLPNO-CCSD(T) and LED calculations were carried out using the aug-cc-pVTZ^75^ (aTZ) and aug-cc-pVQZ^75^ (aQZ) basis sets. Matching auxiliary/C basis sets were used in all cases.^76^ HF and correlation energies computed using aTZ and aQZ basis sets were extrapolated to the estimated complete basis set (CBS) limit using a two-point extrapolation scheme,^77^ as detailed in refs (78, 79). All valence electrons were included in the correlation treatment, as detailed in ref (80). “TightPNO” settings were used in all cases. To reduce the PNO truncation error, energies obtained using *T*_CutPNO_ = 10^–6^ and *T*_CutPNO_ = 10^–7^ settings were extrapolated to the estimated *T*_CutPNO_ = 0 limit, as detailed in ref (63). This approach is denoted as “6/7 PNO extrapolation”.

In particular, the extrapolated energy was computed using the following equation
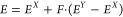
in which *E* is the target correlation energy (for a given basis set) at the complete PNO space, *E*^*X*^ and *E*^*Y*^ are the DLPNO-CCSD(T) correlation energies obtained with *T*_CutPNO_ = 10^–6^ and *T*_CutPNO_ = 10^–7^, respectively, and *F* is a parameter that is set to 1.5. As discussed extensively in ref (63), 6/7 extrapolation calculations are typically 2 times faster than the corresponding *T*_CutPNO_ = 10^–8^ calculations and are also less memory intensive. This indicates that the PNO extrapolation scheme provides a cost-effective alternative to the tightening of the *T*_CutPNO_ threshold.

In LED, LCC-in-HF, and LCC-in-EE calculations, each water molecule was considered as a fragment, unless mentioned otherwise. In the LCC-in-HF methods, the environment was treated at the HF level using various basis sets, namely, aug-cc-pVDZ^75^ (aDZ), cc-pVDZ^75^ (DZ), def2-SVPD (dSVPD),^81^ and def2-TZVPP^81^ (dTZ) basis sets. Optimized structures for water hexamer isomers were obtained from ref (79). In all cases, the perturbative triples contribution (*T*) was calculated using the iterative algorithm.^82^

For the interaction of imidacloprid with the nAChR model, DLPNO-CCSD(T)/LED calculations were carried out using essentially the same computational settings, as described in ref (69). Hence, single point DLPNO-CCSD(T)/dTZ calculations were carried out using matching def2/C auxiliary basis sets. NormalPNO settings were used except that the TCutPairs threshold was set to the TightPNO value of 10^–5^. The RIJCOSX approximation^83,84^ was used in the HF part (the def2/JK basis set was used) with an extremely fine integration grid. In the LCC-in-HF method, the environment was treated at the HF level using the same basis set. The structure of the imidacloprid-nAChR adduct (resistant insect model) was taken from ref (69). As fragments, we selected the imidacloprid ligand, the water molecules (H_2_O), and the W53, W143, R55, Y185, Y192, C187-188, A108, and M14-L122 residues, as described in ref (69).

## 3 Results and Discussion

In this section, the efficiency and accuracy of the LCC-in-HF and LCC-in-EE schemes are tested on water clusters (Section 3.1) and on protein–ligand interactions (Section 3.2).

### 3.1 Water Hexamers

In Section 3.1.1, we initially obtain accurate DLPNO-CCSD(T) reference values for the binding energies of water hexamer clusters. This information is used in Section 3.1.2 to test the accuracy of the LCC-in-HF and LCC-in-EE schemes.

#### 3.1.1 Optimal DLPNO-CCSD(T) Settings for Water Clusters

Second order Møller–Plesset perturbation theory (MP2) and CCSD(T) have been extensively used to study small water clusters.^79^ MP2 is known to slightly underestimate many-body interaction energies in these systems,^79,85−87^ and in fact the deviation between MP2 and CCSD(T) results increases with the size of the cluster.^79^ In this section, we provide the optimal DLPNO-CCSD(T) settings for the calculation of binding energies (see eq 1) of water clusters using canonical CCSD(T) results as the reference. As a first prototype case study, we consider the six isomers of the hexamer water cluster, shown in Figure S1.

The canonical- and DLPNO-CCSD(T) binding energies obtained with different computational settings are shown in Table 1. As previously shown for other water clusters,^62,63^ the dependence of the DLPNO-CCSD(T)/CBS results on the value of the *T*_CutPNO_ threshold appears to be significant. For example, default TightPNO settings (*T*_CutPNO =_ 10^–7^) give a mean absolute error (MAE) of 0.6 kcal/mol with respect to canonical CCSD(T)/CBS. The MAE becomes even higher if looser PNO thresholds (*T*_CutPNO =_ 10^–6^) are used, reaching up to 1.1 kcal/mol. In contrast, 6/7 PNO extrapolation^63^ reduces the MAE of the DLPNO-CCSD(T) results to 0.3 kcal/mol, providing results with essentially canonical CCSD(T) accuracy. Unless specified otherwise, 6/7 PNO extrapolation will be used for all of the DLPNO-CCSD(T), LCC-in-HF, and LCC-in-EE calculations discussed in the following.

**Table 1:** Interaction Energies (kcal/mol) Calculated with DLPNO-CCSD(T) Using Different *T*_CutPNO_ Thresholds for Six Isomers of the Water Hexamer Cluster^a^

isomer	HF	DLPNO-CCSD(T)/CBS *T*_CutPNO_ 10^–6^	DLPNO-CCSD(T)/CBS *T*_CutPNO_ 10^–7^	DLPNO-CCSD(T)/CBS 6/7 PNO Extr.	canonical CCSD(T)/CBS^b^
prism	–32.32	–47.74	–48.26	–48.64	–49.04
cage	–32.25	–47.46	–47.92	–48.26	–48.73
book	–33.49	–47.32	–47.73	–48.04	–48.36
bag	–32.79	–46.81	–47.21	–47.50	–47.85
cyclic	–34.56	–46.40	–46.78	–47.06	–47.28
boat	–33.47	–45.42	–45.79	–46.06	–46.26
MAE^c^	14.77	1.06	0.64	0.33	

a All energies are extrapolated to the estimated CBS limit using aTZ and aQZ basis sets.

b Taken from ref (85).

c Computed using canonical CCSD(T)/CBS as the reference.

#### 3.1.2 Accuracy of LCC-in-HF and LCC-in-EE

Having established the optimal DLPNO-CCSD(T) settings for quantifying binding energies in water clusters, we now turn to the evaluation of the accuracy of the LCC-in-HF and LCC-in-EE approaches. Table 2 shows the HF and correlation components of the binding energy of the prism isomer obtained with different methods and computational settings. As it will be demonstrated numerically below, the main results obtained for this system remain valid for all of the water clusters examined here.

**Table 2:** Binding Energies (kcal/mol) for the Prism Isomer of the Hexamer Water Cluster Calculated Using Embedding MBE Approaches Described in Section 2.3^a^

	Δ*E* (HF)	Δ*E* (Corr)	Δ*E*
DLPNO-CCSD(T)/CBS	–32.32	–16.32	–48.64
LCC(CBS)-in-HF(CBS)	–32.32	–16.38	–48.70
two-body DLPNO-CCSD(T)/CBS^b^	–22.73	–16.69	–39.42
LCC(CBS)-in-HF(aDZ)	–32.33	–16.70	–49.03
LCC(CBS)-in-HF(DZ)	–33.55	–16.60	–50.05
LCC(CBS)-in-HF(dSVPD)	–32.32	–16.54	–48.86
LCC(CBS)-in-EE1(CHELPG^c^/aDZ)	–27.43	–17.00	–44.43
LCC(CBS)-in-EE1(NPA/aDZ)	–29.77	–17.05	–46.82
LCC(CBS)-in-EE1(NPA/aDZ*1.16)	–32.21	–17.10	–49.31
LCC(CBS)-in-EE2(NPA/aDZ*1.16)	–30.60	–17.07	–47.66
LCC(CBS)-in-EE2(NPA/aDZ*1.25)	–31.85	–17.09	–48.94

a Correlation energies were extrapolated to the estimated PNO limit using 6/7 extrapolation.

b Δ*E*^2^ in eq 3.

c CHELPG atomic charges were used, see ref (88) for details.

The overall DLPNO-CCSD(T)/CBS binding energy Δ*E* in this cluster amounts to −48.64 kcal/mol, while the contribution from the two-body component (Δ*E*^2^ in eq 3) is only −39.42 kcal/mol. Hence, many-body effects increase the stability of this system by ∼10 kcal/mol. In-depth information into the chemical origin of such effects can be obtained by analyzing the LED interaction maps (see Section 2.3). This analysis is reported in the SI.

Remarkably enough, the LCC(CBS)-in-HF(CBS) method essentially demonstrates the same accuracy as the parent DLPNO-CCSD(T)/CBS method. In fact, the HF contribution to the binding energy is not affected by the use of an HF embedding (by definition), while the error introduced in the correlation contribution to the binding energy amounts to only 0.06 kcal/mol. These results demonstrate that the use of an HF environment is a cost-effective strategy for computing NCIs between pairs of monomers at the DLPNO-CCSD(T) level in large and complex systems. In particular, the accuracy of the HF embedding proposed here appears to be similar to that obtained using projection-based embedding techniques on similar systems.^50^

Interestingly, if the basis set used for describing the environment is reduced to aDZ, DZ, and dSVPD, the error in the HF component of the binding energy becomes 0.01, 1.22, and 0.00 kcal/mol, respectively. The corresponding errors in the correlation binding energies become 0.38, 0.28, and 0.22 kcal/mol. For comparison, the error obtained in the HF and correlation components of the binding energy by truncating the MBE to the two-body terms (without embedding) is 9.59 kcal/mol and 0.36, respectively. These results demonstrate (i) the importance of including diffuse basis functions in the HF environment to recover mean-field many-body effects accurately, (ii) and the inherent difficulty of describing many-body correlation effects accurately using embedding approaches. In fact, the error in the correlation binding energy in the presence of an HF embedding with a reduced basis set is similar to that obtained by neglecting many-body correlation effects entirely.

**Figure 1:**
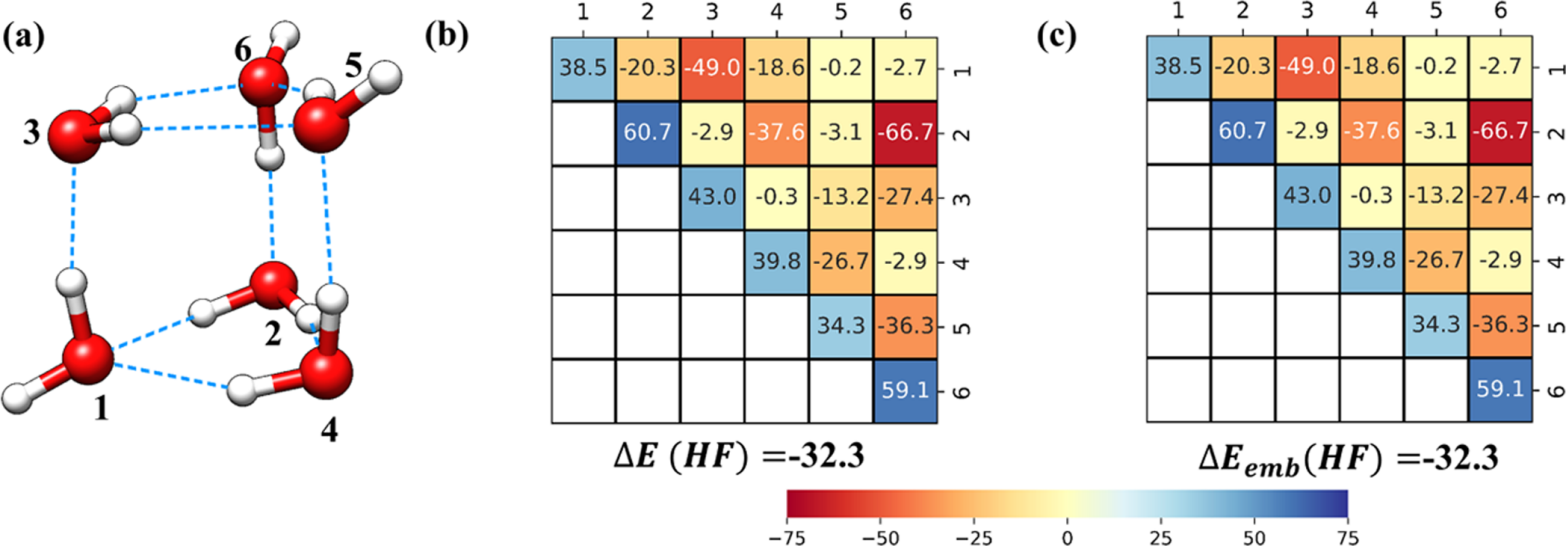
HF component of the LED interaction maps associated with the H-bonding interactions in the (a) prism isomer of the water hexamer; (b) decomposition of the HF-binding energy according to eq 8; and (c) decomposition of the HF-binding energy obtained at the LCC(CBS)-in-HF(aDZ) level, according to eq 14. The color code is shown at the bottom of the figure, with all values in kcal/mol. Bluish colors indicate repulsive interactions, while orange/red indicate attractive interactions.

**Figure 2:**
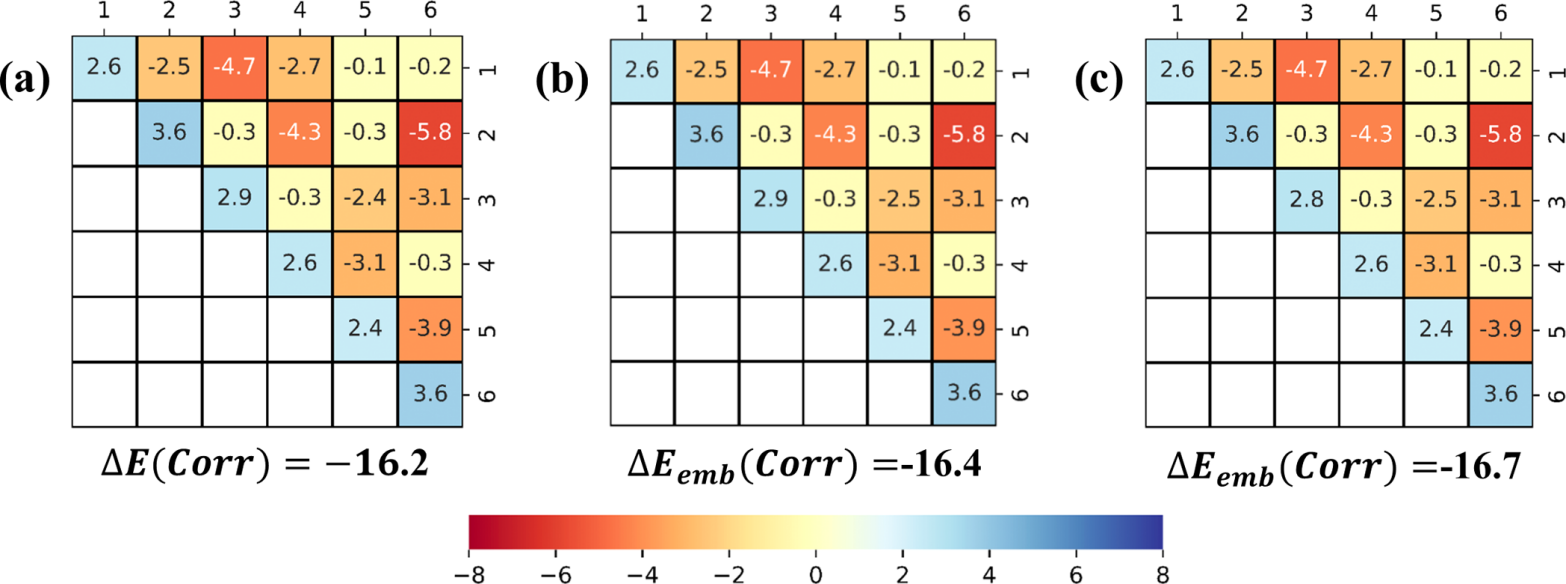
Correlation components of the LED interaction maps associated with the H-bonding interactions in the prism isomer of the water hexamer shown in Figure 1a. (a) Decomposition of the DLPNO-CCSD(T) correlation binding energy,^89^ according to eq 8; (b) decomposition of the LCC(CBS)-in-HF(CBS) correlation binding energy, according to eq 14; and (c) decomposition of the LCC(CBS)-in-HF(aDZ) correlation binding energy, according to eq 14. The color code is shown on the right side, with all values in kcal/mol. Bluish colors indicate repulsive interactions, while orange/red indicate attractive interactions.

Importantly, the LED interaction maps for the HF and correlation components of the binding energy (Figures 1b,c and 2a–c, respectively) show that the LCC-in-HF method not only provides accurate binding energies but also accurate electronic preparation and fragment pairwise interaction energies. Hence, the LED interaction maps demonstrate that the accuracy of this approach is not due to error cancellation but to the accurate description of the entire system of NCIs that contribute to the stability of this complex. This type of analysis appears to be particularly important for the development of novel embedding schemes.

It is now instructive to compare the results just discussed with those obtained using the LCC-in-EE approach defined in Section 2.5. Consistent with previously published results using similar schemes, the accuracy of the mean-field component from such electrostatically embedded methods depends on the actual point charges used for the embedding. In contrast, the correlation binding energy is not affected by the specific point charges used. However, the errors in the correlation binding energy are consistently larger than those obtained with the HF embedding. These results suggest that a more sophisticated HF embedding should be preferred when many-body correlation effects are expected to play an important role.

In our example, accurate mean-field binding energies can be obtained using LCC(CBS)-in-EE1(NPA/aDZ*1.16), in which the charges on the oxygen and hydrogen atoms are on average ca. −1.2 and +0.6, respectively. The LCC-in-EE2(NPA/aDZ*1.16) scheme uses slightly smaller charges (on average, −1.1 and +0.5 for oxygen and hydrogen atoms, respectively), which causes the error on the mean-field binding energy to increase. More accurate values can be obtained by increasing the scaling factor to 1.25. The resulting LCC(CBS)-in-EE2(NPA/aDZ*1.25) scheme provides binding energies of accuracy comparable to that of LCC(CBS)-in-EE1(NPA/aDZ*1.16).

As mentioned above, the results just discussed remain valid for all of the isomers of the water hexamers, as shown in Table 3 (note that the LCC-in-EE2 results were not computed for all systems because they are expected to be similar to those obtained at the LCC-in-EE1 level).

**Table 3:** Binding Energies (kcal/mol) Calculated with Different Approaches for Six Isomers of the Water Hexamer Cluster

isomers	LCC(CBS)-in-HF(CBS)	LCC(CBS)-in-HF(aDZ)	LCC(CBS)-in-EE1(NPA/aDZ*1.16)
prism	–48.70	–49.03	–49.31
cage	–48.36	–48.58	–48.51
book	–48.07	–48.27	–48.72
bag	–47.51	–47.80	–47.49
cyclic	–47.12	–47.38	–48.60
boat	–46.10	–46.41	–47.33
MAE^a^	0.05	0.32	0.74
MAE(HF)^b^	0.00	0.12	0.68
MAE(Corr.)^c^	0.05	0.19	0.39

a MAE computed using as reference DLPNO-CCSD(T)/CBS results (see Table 1).

b MAE obtained for the mean-field component of the energy.

c MAE obtained for the correlation component of the energy using HF/CBS as the reference.

Being embarrassingly parallel, the computational cost of LCC-in-HF and LCC-in-EE calculations is limited by that of the largest dimer considered, provided that enough computational resources are available. In the present case, all dimers have a similar computational cost. For example, the energy calculation for an embedded water dimer at the LCC(aQZ)-in-EE2(NPA/aDZ*1.16) level (*T*_CutPNO_ 10^–7^) took on average 0.13 h using four cores from a single cluster node equipped with Intel Xeon E5-2690v2 CPUs and 64 GB of RAM (the SCF part of the calculation took only a few seconds). In contrast, a DLPNO-CCSD(T)/aQZ calculation on the “Bag” isomer took 4 h using the same computational resources (only 4 min were required for the SCF part of the calculation). For comparison, the coupled cluster part of LCC(aQZ)-in- HF(aDZ) and LCC(aQZ)-in-HF(aQZ) calculations on the embedded dimers using the same DLPNO settings took 0.13 and 0.5 h, respectively.

**Figure 3:**
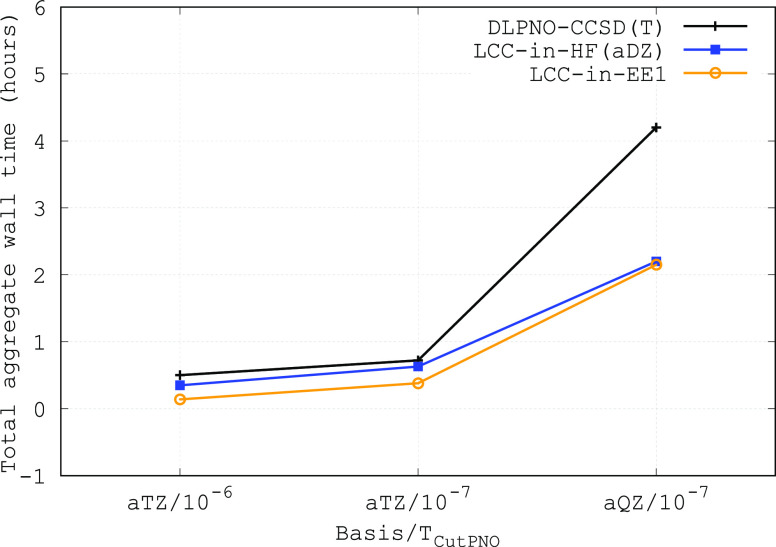
Total aggregate wall time required to compute the energy of the Bag isomer with various computational settings. All calculations were carried out using four cores from a single cluster node equipped with Intel Xeon E5-2690v2 CPUs and 64 GB of RAM.

The total aggregate wall time required to compute the energy of the Bag isomer with various computational settings is shown in Figure 3. For the purpose of this comparison, all calculations were run sequentially on a single node with four cores, meaning that we did not exploit the embarrassingly parallel nature of the LCC-in-EE and LCC-in-HF methods. For example, timings could be trivially reduced by running the individual embedded dimer calculations on separate nodes. Nevertheless, LCC-in-EE and LCC-in-HF are still more efficient than their parent DLPNO-CCSD(T) method, and the efficiency difference increases with large basis sets. It is also worth mentioning that for this system, LCC-in-EE1 and LCC-in-HF show similar performances. This is due to the fact that the computational cost in this case is dominated by the coupled cluster part, which is similar for both methods. However, for larger systems, LCC-in-EE1 will become more efficient than LCC-in-HF, as the former does not require to perform HF calculations on the supersystem. This makes LCC-in-EE1 and LCC-in-EE2 particularly promising for the study of condensed phase systems.

### 3.2 Protein–Ligand Interactions

**Figure 4:**
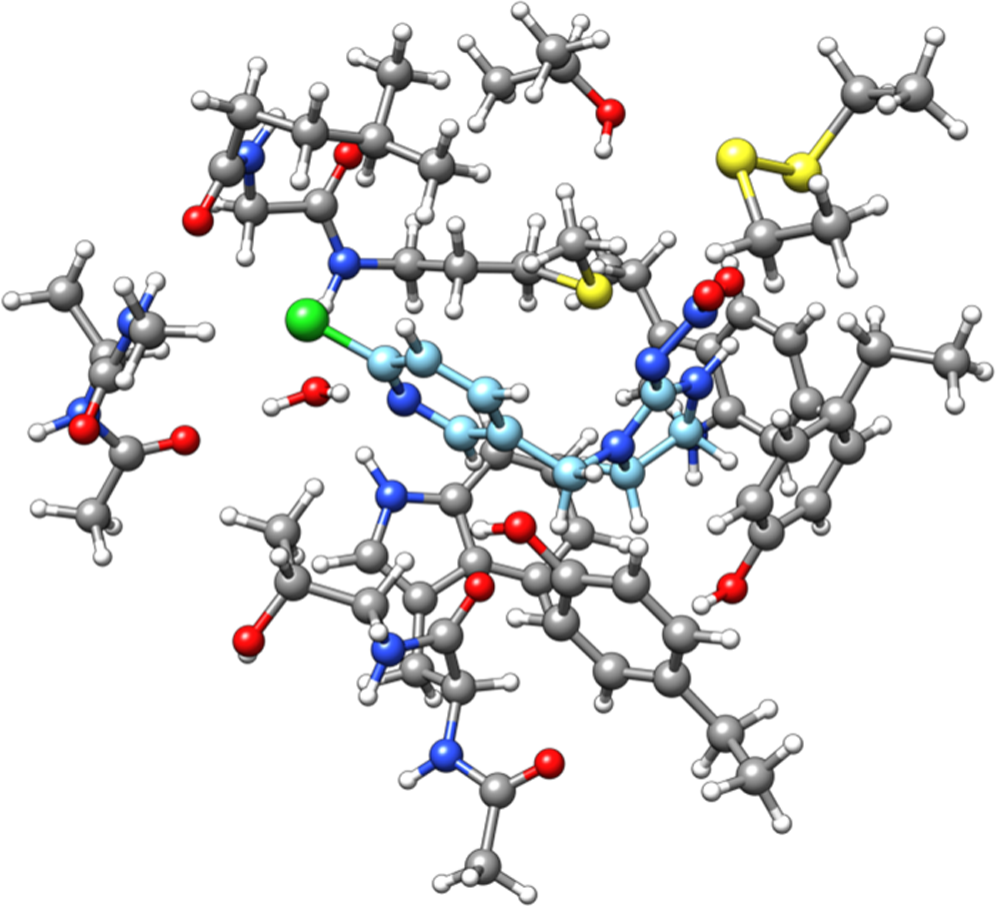
Cluster model for the binding of the imidacloprid ligand to a nAChR model. The system has 223 atoms. The structure is taken from ref (69). Carbon atoms of the imidacloprid ligand are shown in light blue color.

In this section, the efficiency and the accuracy of the LCC-in-HF and LCC-in-EE schemes are discussed on a model system representing the interaction of the imidacloprid ligand with the active site of the nicotinic acetylcholine receptor (nAChR).^90^ Imidacloprid is a neonicotinoid synthetic insecticide and it is important to study its interaction with insect nAChRs to understand the selective toxicity of these types of insecticides.^91^ The structure of the imidacloprid-nAChR adduct is shown in Figure 4.^69^

The total energy of the system computed at the DLPNO-CCSD(T)/dTZ, LCC(dTZ)-in-HF(dTZ), and LCC(dTZ)-in-EE2(NPA/aDZ*1.25) levels is shown in Table 4. In the LCC-in-HF and LCC-in-EE case, the total energy was computed using eq 15 (see Section 2.6 for the fragment definition).

**Table 4:** Total Energy *E*_tot_, HF energy *E*_HF_, and Correlation Energy *E*_cor_ (in Hartree) for the Imidacloprid-nAChR Adduct Shown in Figure 4 Computed at the DLPNO-CCSD(T)/dTZ Level of Theory, as Described in Section 2.6^a^

	DLPNO-CCSD(T)/dTZ	LCC(dTZ)-in-HF(dTZ)	LCC(dTZ)-in-EE2(NPA/aDZ*1.25)
	energy (Hartree)	Δ (kcal/mol)	Δ (kcal/mol)
*E*_HF_	–6281.59409	0.00	1.40
*E*_cor_	–20.4988543	–0.11	–0.95
*E*_tot_	–6302.09294	–0.11	0.45

a Δ (in kcal/mol) is the deviation obtained for the same energy contributions computed at various levels of theory with respect to the DLPNO-CCSD(T)/dTZ reference.

For this system, all methods reproduce the total DLPNO-CCSD(T) energy extremely well, with an error of only −0.11 and 0.45 kcal/mol for LCC(dTZ)-in-HF(dTZ) and LCC(dTZ)-in-EE2(NPA/aDZ*1.25), respectively. In the LCC(dTZ)-in-EE2(NPA/aDZ*1.25) case, the error in the mean-field component of the energy amounts to 1.40 kcal/mol and it partially cancels out with the −0.95 kcal/mol error observed in the correlation energy.

To determine whether or not this remarkable accuracy is the result of error cancellation between different interaction terms, we examined the individual errors associated with the key interactions responsible for the binding of the ligand to the protein, *i.e*., those between the ligand and the residues and in the active site.^69^ In the DLPNO-CCSD(T) case, these are quantified by the *E*_*XY*_^int^ terms appearing in eq 7, while in embedded approaches these are given by the *E*_*XY*_^int,2^(***S***) terms in eq 15.

The comparison between the *E*_*XY*_^int^ and *E*_*XY*_^int,2^(***S***) terms associated with the same *XY* dimer (Table 5) revealed that the error observed in the individual interaction terms is of the same order of that observed for the total energy of the system. Thus, the difference between *E*_*XY*_^int^ and *E*_*XY*_^int,2^(***S***) is relatively small, reaching a maximum of 0.14 kcal/mol with LCC(dTZ)-in-HF(dTZ) and of 1.00 kcal/mol with LCC(dTZ)-in-EE2(NPA/aDZ*1.25). For comparison, the deviation that one would obtain without any embedding (*E*_XY_^int,2^ – *E*_*XY*_^int^) is significantly larger, reaching up to 4.2 kcal/mol for the imidacloprid-W53 interaction. Hence, the LCC-in-HF and LCC-in-EE methods provide the right results for the right reasons, even though the calculation of total energies benefit from favorable error compensation between the terms in eq 15.

**Table 5:** LED Interactions *E*_*XY*_^int^ between the Imidacloprid Ligand and the Residues in the Active Site of the “Resistant” nAChR Model at the DLPNO-CCSD(T)/def2-TZVPP Level^a^

	DLPNO-CCSD(T)/dTZ	LCC(dTZ)-in-HF(dTZ)		LCC(dTZ)-in-EE2(NPA/aDZ*1.25)	
residue	*E*_*XY*_^int^ (kcal/mol)	Δ (kcal/mol)	wall time^b^ (h)	Δ (kcal/mol)	wall time (h)
H20	–42.80	–0.09	4.2	–0.39	0.7
W53	–22.94	–0.05	5.1	–0.01	2.3
W143	–70.65	–0.14	12.0	–1.00	5.5
R55	–0.48	0.00	4.2	0.02	0.9
Y185	–48.43	–0.10	6.8	–0.39	3.4
Y192	–19.40	–0.08	4.5	–0.32	2.2
C187-188	–18.89	–0.03	4.8	–0.48	1.1
A103	–5.90	–0.05	8.2	–0.44	1.3
M14-L112	–65.61	–0.16	4.2	–0.51	3.9

a Δ (in kcal/mol) is computed as *E*_*XY*_^int,2^(***S***)- *E*_*XY*_^int^, where *E*_*XY*_^int,2^(***S***) is obtained using different embedding schemes. For LCC-in-HF and LCC-in-EE2, the wall time for computing the energy of the corresponding (embedded) dimers is reported in hours.

b Wall time for the HF/dTZ calculation on the full protein–ligand adduct is 4.5 h. In the LCC(dTZ)-in-HF(dTZ) scheme, this calculation needs to be carried out only once. The resulting orbitals are used to compute all of the terms in the MBE.

In terms of efficiency, a DLPNO-CCSD(T) single point energy calculation on the whole system required 64 h using 12 cores from a single cluster node equipped with Intel Xeon E5-2687Wv4 CPUs and 256 GB of RAM. In contrast, the LCC(dTZ)-in-HF(dTZ) and LCC(dTZ)-in-EE2(NPA/aDZ*1.25) results were obtained from separate calculations, and the associated wall times are also reported in Table 5. In the present case, in which 12 cores were assigned to each subsystem, the most expensive dimer calculations took 12.0 and 5.5 h for LCC(dTZ)-in-HF(dTZ) and EE2(NPA/aDZ*1.25), respectively.

Finally, it is worth emphasizing that in some chemical applications, one is only interested in the quantification and analysis of selected interactions at the coupled cluster level. In this case, the approaches introduced here appear to be particularly promising, as they allow us to focus the computational resources on the subsystem of interest. For example, they can be used to monitor how the interaction between the ligand and a specific residue or solvent molecule changes in various snapshots of a molecular dynamic simulation, without the need to treat the whole system at the coupled cluster level.

## 4 Conclusions

We have introduced the LCC-in-HF and the LCC-in-EE methods for the quantification and analysis of NCIs in molecular aggregates at the local coupled cluster level. These methods were defined by combining two different expansions of the DLPNO-CCSD(T) energy, *i.e*., the MBE and the LED. In the LCC-in-HF scheme, the one- and two-body terms in the MBE are computed in the presence of an HF embedding, while in the LCC-in-EE scheme, the environment is replaced by point charges. In both cases, the LED scheme was used to decompose the terms in the embedded MBE into contributions from:

(1) the embedded monomers;
(2) the interaction energy between pairs of embedded monomers (for the terms of order higher than 1)
(3) the energy of the environment; and
(4) the interaction energy between the environment and each of the monomers.

This information is used to determine the total energy of the supersystem, while providing at the same time quantitative information into the underlying pattern of NCIs as well as their cooperativity effects.

Both approaches were tested on a series of water clusters and on the adduct between the imidacloprid ligand and a nAChR model. It was found that the LCC-in-HF scheme essentially retains the same accuracy of the parent DLPNO-CCSD(T) method, provided that a sufficiently large basis set is used in the HF embedding. In contrast, the accuracy of the LCC-in-EE approach depends on the actual charges used for the embedding. For the systems considered in this work, chemical accuracy can be achieved using NPA charges computed at the HF level for the individual fragments and scaled by a suitable factor. Notably, the use of the more sophisticated HF embedding is recommended for those systems in which many-body correlation effects are expected to be especially important.

Finally, the LCC-in-HF and the LCC-in-EE schemes are embarrassingly parallel and require less memory than their parent DLPNO-CCSD(T) method. Hence, they appear to be particularly promising for the study of large and complex systems in the condensed phase.

## Supporting Information Available

Analysis of cooperative effects for the prism isomer of the water hexamer and XYZ structures (PDF)
