# Supplementary material for: Fragment-Based Local Coupled Cluster Embedding Approach for the Quantification and Analysis of Noncovalent Interactions: Exploring the Many-Body Expansion of the Local Coupled Cluster Energy
Source: J Chem Theory Comput. 2021 May 26;17(6):3348–59. doi: 10.1021/acs.jctc.1c00005 (PMC8190956; doi:10.1021/acs.jctc.1c00005)
Supplement: Supplementary file 1 — ct1c00005_si_001.pdf [file ct1c00005_si_001.pdf]

SUPPORTING INFORMATION

Fragment-Based Local Coupled Cluster Embedding Approach for the Quantification and Analysis of Noncovalent Interactions: Exploring the Many Body Expansion of the Local Coupled Cluster Energy

Soumen Ghosh, Frank Neese, Robert Izsàk, Giovanni Bistoni*

Max-Planck-Institut für Kohlenforschung, Kaiser-Wilhelm-Platz 1, D-45470 Mülheim an der Ruhr, Germany

ANALYSIS OF COOPERATIVITY EFFECTS: THE PRISM ISOMER S2

XYZ STRUCTURES S7

REFERNCES S11

ANALYSIS OF COOPERATIVITY EFFECTS: THE PRISM ISOMER

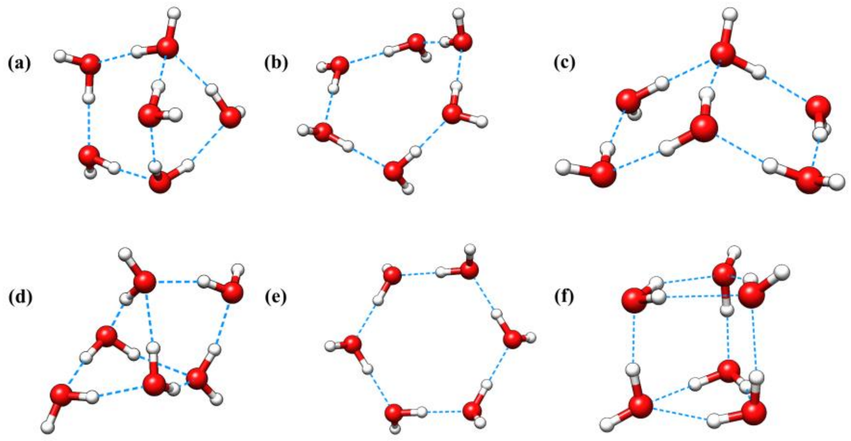

Figure S1 Structure of different hexamer water clusters

As a first example, we consider the prism isomer of the hexamer water cluster, which is the most stable isomer for this system. Fig. S2a shows the breakdown of ΔE into fragment-pairwise contributions, while the decomposition of ΔE^2 is shown in Fig. S2b. In the LED maps, diagonal elements represent electronic preparation energies of different water molecules and off-diagonal elements represent pair-wise interaction energies between two water molecules.

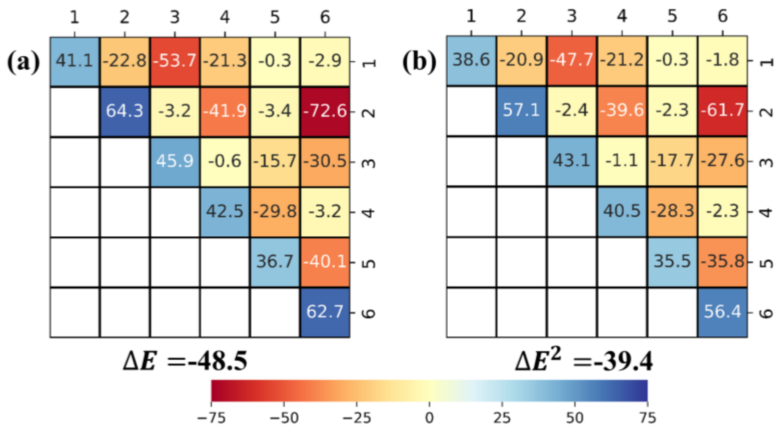

Figure S2. LED interaction map for the H-bonding interactions in the prism isomer shown in Fig. S1. (a) decomposition of ΔE, (b) decomposition of ΔE^2. The color scale for the heat maps is shown in the bottom, with all values in kcal/mol. Bluish colors indicate repulsive interactions, while orange/red indicate attractive interactions.

This figure provides a clear-cut visual representation of the various noncovalent interactions that contribute to the stability of this system. The electronic preparation is largest for molecules **2** and **6**, whilst all other fragments show significantly lower and similar electronic preparation energies. Consistent with this picture, the interaction between molecule **2** and **6** is the strongest among all the water pairwise interactions in this system. The second strongest interaction is found between molecules **1** and **3**. Hence, it can be concluded that the strongest interactions occur when a water molecule that has both lone pairs involved in H-bonds donates one of its protons to a water molecule that has both protons involved in H-bonds. Qualitatively, the LED interaction map associated to $\Delta E^2$is similar to the one just discussed, but with two important differences: (i) the electronic preparation is generally less repulsive; (ii) pairwise interactions are less attractive.

To clearly show cooperativity effects on these Hydrogen-bonding interactions, the difference between Fig. S2a and Fig. S2b is shown in Fig. S3a. Hereafter, this type of visual representation is called “LED cooperativity map”. The sum of all the elements in Fig. S3a gives ΔE_{coop}. Fig. S3b and Fig. S3c show the LED cooperativity maps associated with the HF and correlation components of ΔE_{coop}., respectively.

From these figures, a few important conclusions can be drawn concerning the origin of cooperativity in this system:

(i) The dominant many-body effects are associated with the mean field contribution of the energy. These results are consistent with previous results from Alken et al.[1] as well as with previous SAPT studies.[2-3,4]

(ii) Cooperativity increases the electronic preparation of water molecules significantly. Hence, the interplay of various interactions in the water hexamer significantly perturbs the electronic structure (*e.g.*, the one electron density) of the water molecules. The Many-body correlation contribution to the electronic preparation is negligible.

(iii) Cooperativity also increases the strength of all pairwise interactions. Although the dominant many body contribution comes from the mean-field component of the energy, many body long-range correlation effects on the fragment-pairwise interactions are not negligible and overall repulsive.

(iv) Qualitatively speaking, mean-field cooperativity originates from the fact that the hydrogen bonding donor atom becomes partially more negative upon formation of an H-bond, whilst the acceptor atom becomes partially more positive. This is consistent with the fact that the **2-6** and **1-3** interactions discussed above are also the interactions with the strongest many-body contributions.

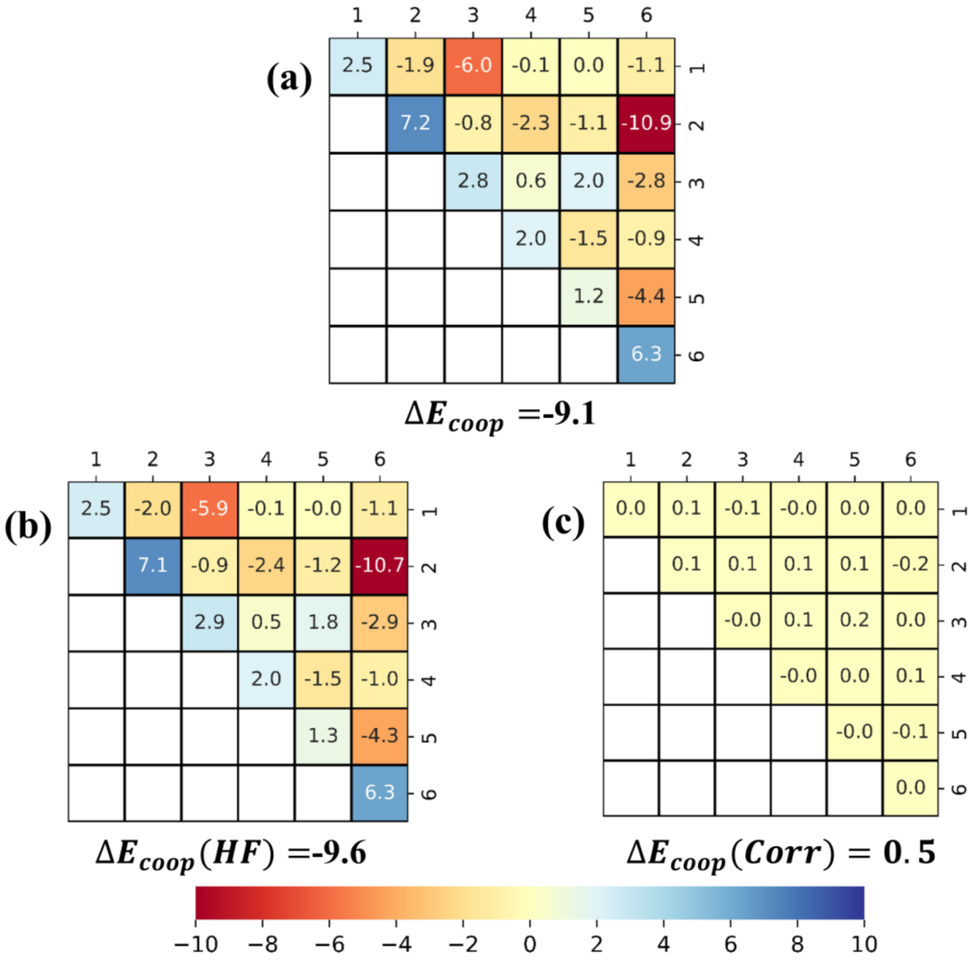

Figure S3. LED cooperativity maps for the H-bonding interactions in the prism isomer shown in Fig. 1. (a) LED map of $\Delta E_{coop.}$. (b) LED map for the HF component of $\Delta E_{coop.}$ (c) LED map for the correlation component of $\Delta E_{coop.}$. The color scale for the heat maps is shown on the right side, with all values in kcal/mol. Bluish colors indicate repulsive interactions, while orange/red indicate attractive interactions. All energies are extrapolated to the estimated CBS limit using aTZ and aQZ basis sets.

Finally, cooperativity effects can also be divided into electronic preparation, exchange, electrostatics, dispersion and non-dispersion inter-fragment correlation contributions, as shown in Fig. S4.

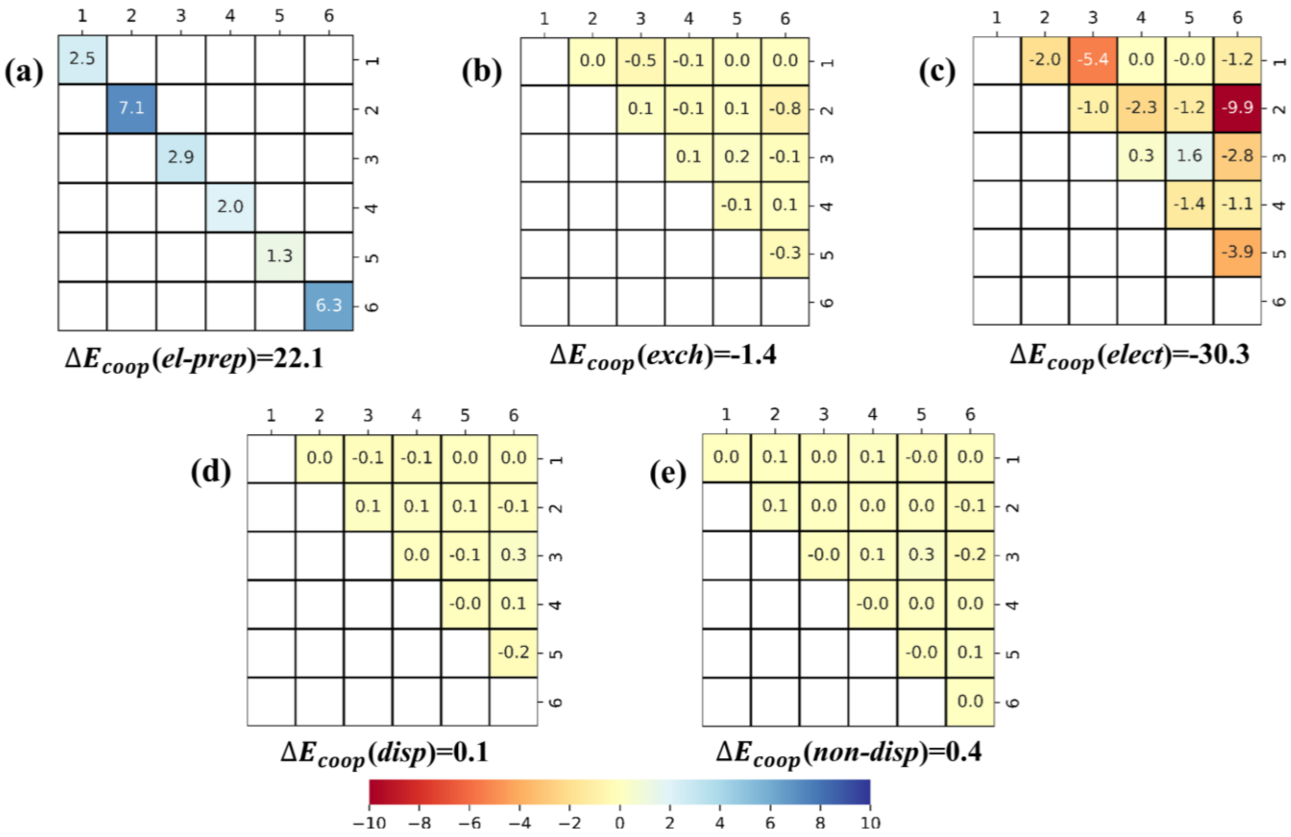

Figure S4. LED cooperativity maps for different LED components to the binding energy of the water hexamer prism isomer. (a) LED map for HF electronic preparation, (b) LED map for HF exchange energy, (c) LED map for the HF electrostatic energy, (d) LED map for dispersion interactions and (e) LED map for non-dispersion correlation interactions. The color scale for the heat maps is shown on the right side, with all values in kcal/mol. Bluish colors indicate repulsive interactions, while orange/red indicate attractive interactions. All energies are extrapolated to the estimated CBS limit using aTZ and aQZ basis sets.

XYZ STRUCTURES

Isomers of hexamer water cluster

Bag

O	3.262742032	-0.454795268	4.240469034
H	2.865107792	-0.451357938	5.114437088
H	3.115718785	-1.364305069	3.876425220
O	2.840014385	-2.814725454	3.050776767
H	3.390269031	-2.809795280	2.247292133
H	1.935814798	-2.790059609	2.691199645
O	4.034394599	-2.381407422	0.511017529
H	4.002861636	-3.066459697	-0.162496008
H	3.360878586	-1.730458042	0.245868765
O	2.850589788	1.222357044	2.198366926
H	3.700360330	1.653090752	2.068860912
H	2.973369979	0.669207248	3.007903025
O	1.992436285	-0.427900679	0.290257317
H	2.315737029	0.235130643	0.954455326
H	1.709654962	0.083909567	-0.472956316
O	0.435132186	-2.349603554	1.614161903
H	-0.399257087	-2.018006636	1.956416696
H	0.824660055	-1.606677606	1.117690489

Boat

O	-1.753042425	0.416462959	0.356105280
H	-1.120933301	-0.183098298	0.812596940
H	-2.593310567	-0.048459739	0.359102074
O	2.482542995	-1.248988714	0.449488905
H	2.616828786	-1.798226083	-0.326968465

H	2.828107886	-0.362220961	0.204648083
O	0.012631031	-1.241255898	1.573836894
H	0.908618001	-1.252949881	1.168524322
H	0.168950409	-1.135399007	2.515952529
O	3.364853284	1.252522284	-0.119963272
H	2.731853532	1.891821037	-0.517486897
H	4.212012919	1.443946099	-0.529713339
O	-0.845377446	1.902266646	-1.724481185
H	-1.198971324	1.335278306	-1.003913817
H	-0.953209570	1.388839038	-2.529056860
O	1.597560181	2.978636709	-1.236245995
H	1.417961949	3.808511891	-0.786488687
H	0.711446199	2.593888593	-1.420550970

Book

O	2.379124546	0.832605391	-1.165013998
H	2.694709407	0.367893670	-1.944580755
H	2.495987885	0.198615323	-0.417438154
O	-2.267483285	-1.770995490	0.333961948
H	-2.364616139	-1.216972516	-0.465985472
H	-3.140260734	-1.787813796	0.735691831
O	2.354243565	-0.876469943	0.906725358
H	2.874210883	-0.769017001	1.707363793
H	1.414695939	-0.742989268	1.182009739
O	-0.202353616	-0.237891540	1.424521204
H	-0.917957915	-0.851263432	1.167148157
H	-0.308249314	0.507709847	0.814157731
O	-2.241748192	0.013264486	-1.781940081

H	-2.094607514	-0.256612088	-2.692236504
H	-1.501342097	0.612898758	-1.565728288
O	-0.167787702	1.601628594	-0.846870354
H	-0.202223113	2.562381171	-0.838467890
H	0.771290368	1.367487853	-1.048937437

Cage

O	-0.728171453	-1.001938273	-1.293324355
H	-1.457175360	-1.462613673	-1.717877807
H	-0.768285764	-1.241551183	-0.330105003
O	-2.193230907	0.800363894	1.803255475
H	-2.407265376	1.418166857	2.506597245
H	-1.652516736	1.301606319	1.155801295
O	-0.539033186	-1.371554122	1.344931448
H	-1.165095025	-0.719744877	1.715283491
H	0.324999315	-0.942877517	1.448948311
O	1.825475901	0.224963492	0.821073287
H	2.077332444	-0.222124844	-0.016931705
H	2.647514448	0.520903138	1.221744692
O	-0.483016170	1.703129780	-0.145801169
H	-0.688546723	1.011190769	-0.792430040
H	0.379705985	1.420649751	0.199171755
O	1.994756464	-1.013109360	-1.591644710
H	2.364879446	-1.888454222	-1.735052429
H	1.025244068	-1.125998317	-1.667905262

Cyclic

O	-1.850461250	2.234155785	-0.314000263
H	-2.234491542	2.510430390	-1.150103926
H	-2.232556303	1.345090787	-0.138214269
O	0.809710106	2.588501466	0.048265549
H	-0.154677670	2.453026132	-0.089328120
H	0.873757740	3.186385462	0.797470974
O	1.367067367	-2.021647245	0.464245600
H	1.751131298	-2.297924419	1.300333633
H	1.749175739	-1.132591533	0.288439779
O	2.435152857	0.423097975	-0.000202719
H	1.840030323	1.205757637	0.024308276
H	2.971002928	0.541090533	-0.788758061
O	-1.293114344	-2.376004852	0.102006992
H	-1.357139935	-2.973879373	-0.647206589
H	-0.328735431	-2.240520685	0.239646648
O	-2.918550905	-0.210593224	0.150433740
H	-3.454408941	-0.328571863	0.938984096
H	-2.323419946	-0.993246104	0.125939700

Prism

O	0.041948971	-2.058573616	-0.411705886
H	-0.749504423	-1.476896725	-0.371632667
H	-0.294603829	-2.958750744	-0.399359741
O	1.690178787	-0.039257978	-1.709436451
H	1.974517606	0.001266283	-0.772889797
H	1.230242616	-0.891730929	-1.724042211
O	-1.903794739	-0.140389401	-0.301836475
H	-1.593576791	0.486317154	-0.975557170

H	-1.731877237	0.347010919	0.515828171
O	1.781999366	-0.233440242	1.066446660
H	1.238894986	-1.018268120	0.897519153
H	1.136235100	0.453009461	1.297721636
O	-0.281628273	1.812439584	1.159731637
H	-0.277135360	2.630566975	1.664501696
H	-0.214557050	2.073552047	0.220659084
O	-0.233113802	1.782690290	-1.602807998
H	0.519532374	1.148403791	-1.752530352
H	-0.205381112	2.411251760	-2.329337500

REFERENCES

1. Alkan, M.; Xu, P.; Gordon, M. S., Many-Body Dispersion in Molecular Clusters. *The Journal of Physical Chemistry A* **2019**, *123*, 8406-8416.
2. Milet, A.; Moszynski, R.; Wormer, P. E.; van der Avoird, A., Hydrogen Bonding in Water Clusters: Pair and Many-Body Interactions from Symmetry-Adapted Perturbation Theory. *The Journal of Physical Chemistry A* **1999**, *103*, 6811-6819.
3. Heßelmann, A., Correlation Effects and Many-Body Interactions in Water Clusters. *Beilstein journal of organic chemistry* **2018**, *14*, 979-991.
4. Podeszwa, R.; Szalewicz, K., Three-Body Symmetry-Adapted Perturbation Theory Based on Kohn-Sham Description of the Monomers. *The Journal of chemical physics* **2007**, *126*, 194101.
